# Natural Biodiversity Breaks Plant Yield Barriers

**DOI:** 10.1371/journal.pbio.0020331

**Published:** 2004-08-24

The birth of agriculture, some 10,000 years ago in the Middle East's Fertile Crescent, revolutionized human culture and society. Refined farming techniques led to increased yields and freed humans from the demands of constant foraging. Along with that freedom came social complexity, division of labor, improved standards of living, and a measure of leisure time. Agriculture also led to overpopulation followed by starvation, conflict over fertile farming land, and environmental damage. For the Maya and other civilizations, such consequences proved fatal.

Many consumer and environmental groups believe that modern industrial agricultural practices like factory farming of animals and genetic engineering of crops threaten to bring similar ruin. But with 6 billion people living on the planet—a figure that's expected to increase 50% in just 50 years—many plant scientists believe that feeding a burgeoning population will require the tools of biotechnology. Plant breeders face the daunting challenge of developing high-yielding, nutritious crops that will improve the global quality of life without harming the environment or appropriating dwindling natural habitats for agricultural production. A major roadblock to feeding the world is a continuing decline in the genetic diversity of agricultural crops, which has in turn limited their yield improvement. (Domestication often involves inbreeding, which by definition restricts the gene pool.) Now Amit Gur and Dani Zamir of Hebrew University report a way to lift these productivity barriers by tapping into the natural diversity of wild plants.

Traditional plant breeders improve the quality and yield of crops by crossing plants with desired traits to create a new, hopefully improved, hybrid strain. But traditional breeding is limited by the available gene pool of a cultivated plant species and eventually hits a wall—reshuffling the same genetic variation can boost yield only so much. With the advent of biotechnology, plant scientists were buoyed by the prospect of improving plants through genetic modification. But aside from a few successes with introducing single-gene herbicide- and pest-resistant traits, most plant traits have proved too complex to repay the incorporation of a single transgene—that is, a gene taken from a different species—with the hoped-for response. Biotech-based investigations and applications in plant science have also been hampered by consumer reaction against genetically modified organisms. (For more on the techniques of modern plant breeding, see the essay “Diversifying Selection in Plant Breeding,” also in this issue.)

Faced with these limitations, Gur and Zamir tried another approach—a back-to-nature approach. “Natural biodiversity is an unexploited sustainable resource that can enrich the genetic basis of cultivated plants,” they explain in the report. The distantly related wild cousins of cultivated plants can be seen as a “huge natural mutagenesis resource” with novel gene variants that can increase productivity, quality, and adaptability. Not only that, the genetic material of wild plants—every gene and regulatory element—has already been refined and tested by over a billion years of evolution and natural selection.

To identify genomic regions in wild tomato species that affect yield, Gur and Zamir created a population of hybrid crosses of a wild tomato species and a cultivated tomato species; each line had a single genomic region from the wild tomato inserted into the cultivated plant. Rather than introducing a single wild tomato gene into the cultivated plants, the authors used a “pyramided” strategy that combined three independent yield-enhancing genomic regions from the wild species into the new plant line. Plants were grown over three seasons, during which they were exposed to different environments, including drought. By combining traditional phenotyping techniques—which characterize the plant's physical traits based on its genetic makeup—with genetic marker analysis, the authors identified a number of wild tomato genomic regions that increased yield.

Their results demonstrate that an approach based on biodiversity—which takes advantage of the rich genetic variation inherent in wild relatives of cultivated crops—can produce varieties that outperform a commercially available hybrid tomato in both yield and drought resistance. Gur and Zamir attribute the improved performance to their unique pyramiding strategy. Their hybrid model—applying the tools of modern genomics to traditional plant breeding—offers plant breeders a powerful approach to improving the quality and yield of cultivated plants by taking advantage of the inherent biodiversity of the natural world. It's a strategy that may well apply to rice, wheat, and other vital staples of the world's food supply.

